# Supplementary material for: Composite Uremic Load and Physical Performance in Hemodialysis Patients: A Cross-Sectional Study
Source: Toxins (Basel). 2020 Feb 22;12(2):135. doi: 10.3390/toxins12020135 (PMC7076769; doi:10.3390/toxins12020135)
Supplement: Supplementary file 1 [file toxins-12-00135-s001.pdf]

Supplementary Materials: Composite Uremic Load and Physical Performance in Hemodialysis Patients: A Cross-Sectional Study

Karsten Vanden Wyngaert *, Amaryllis H. Van Craenenbroeck, Els Holvoet, Patrick Calders, Wim Van Biesen and Sunny Eloot

Table S1. Detailed association between relevant associations of uremic toxins and domains of physical performance.

Variable	Handgrip Strength		Quadriceps Strength		STS	
	Estimate (SE)	*p* Value	Estimate (SE)	*p* Value	Estimate (SE)	*p* Value
IS total	−34.89 (12.7)	0.008	−286.28 (123.5)	0.024	45.09 (32.1)	0.166
IS free	61.10 (19.9)	0.003	252.61 (216.6)	0.248	−57.84 (53.3)	0.282
pCS total	30.79 (13.3)	0.024	−199.73 (116.5)	0.308	−15.80 (31.8)	0.621
pCS free	−31.55 (19.8)	0.115	227.41 (198.1)	0.256	20.21 (47.7)	0.673
pCG total	−16.57 (14.8)	0.266	−519.82 (272.9)	0.062	192.42 (76.7)	0.015
pCG free	−15.44 (14.7)	0.298	485.41 (271.1)	0.078	−153.87 (76.4)	0.048
IAA total	46.58 (18.1)	0.012	415.88 (217.0)	0.060	−84.41 (57.3)	0.146
IAA free	−53.97 (23.0)	0.022	−650.84 (297.1)	0.032	134.77 (78.5)	0.015
HA total	19.93 (20.1)	0.326	317.11 (276.7)	0.256	134.94 (73.6)	0.072
HA free	2.09 (21.8)	0.924	−278.93 (306.6)	0.367	−190.98 (81.1)	0.022
CMPF	−7.26 (7.8)	0.358	−5.68 (64.0)	0.930	29.26 (16.6)	0.082
UA	8.99 (7.8)	0.256	−21.89 (65.1)	0.738	−3.39 (16.6)	0.839

Data are presented as estimated beta-values and estimated standard error (SE), the analyses are controlled for age and gender. Abbreviations: STS, sit-to-stand.

Table S2. Functional muscle strength and uremic toxins.

Uremic Toxin Concentrations (mg/dL)	Sit-to-Stand			*p*-Value
	Good (<13 s)	Moderate (13 s–49 s)	Poor (50 s)	
IS total	1.883 [1.341; 2.426]	1.613 [1.108; 2.117]	1.708 [1.259; 2.157]	0.617
IS free	0.121 [0.068; 0.175]	0.143 [0.048; 0.238]	0.132 [0.085; 0.180]	0.589
pCS total	3.307 [2.513; 4.102]	3.252 [2.544; 3.961]	3.640 [2.938; 4.342]	0.753
pCS free	0.237 [0.151; 0.323]	0.280 [0.146; 0.415]	0.292 [0.220; 0.364]	0.278
pCG total	0.215 [0.118; 0.312]	0.351 [0.168; 0.534]	0.355 [0.233; 0.478]	0.278
pCG free	0.200 [0.107; 0.292]	0.328 [0.156; 0.500]	0.319 [0.208; 0.430]	0.328
IAA total	0.157 [0.111; 0.202]	0.177 [0.097; 0.256]	0.157 [0.107; 0.207]	0.925
IAA free	0.048 [0.030; 0.065]	0.065 [0.014; 0.116]	0.054 [0.027; 0.081]	0.934
HA total	2.248 [1.484; 3.012]	2.980 [1.671; 4.288]	2.343 [1.477; 3.210]	0.991
HA free	1.203 [0.756; 1.652]	1.804 [0.874; 2.735]	1.272 [0.769; 1.776]	0.970

CMPF	0.570 [0.299; 0.841]	0.593 [0.390; 0.796]	0.754 [0.436; 1.072]	0.411
UA	6.639 [6.137; 7.140]	6.173 [5.591; 6.756]	6.042 [5.467; 6.616]	0.191

Data are presented as median and confidence interval [CI]; patients were allocated to a good, moderate or poor lower limb muscle function group based on the tertiles of the Sit-to-Stand test, <13 s, from 13 to 49 s, and 50 s or more, respectively.

Table S3. Upper limb muscle strength and uremic toxins.

**Uremic Toxin Concentrations (mg/dL)**	**Handgrip Strength**			***p*-Value**
	**High (>103%)**	**Moderate (83–103%)**	**Low (<83%)**	
IS total	1.509 [1.082; 1.935]	1.792 [1.318; 2.265]	1.924 [1.337; 2.511]	0.519
IS free	0.098 [0.050; 0.146]	0.157 [0.066; 0.249]	0.142 [0.090; 0.195]	0.351
pCS total	3.095 [2.564; 3.621]	3.789 [3.020; 4.557]	3.344 [2.467; 4.221]	0.364
pCS free	0.206 [0.153; 0.259]	0.325 [0.190; 0.460]	0.281 [0.191; 0.372]	0.307
pCG total	0.234 [0.123; 0.346]	0.314 [0.167; 0.461]	0.379 [0.225; 0.532]	0.477
pCG free	0.214 [0.107; 0.320]	0.293 [0.155; 0.432]	0.345 [0.205; 0.485]	0.462
IAA total	0.138 [0.104; 0.172]	0.204 [0.123; 0.285]	0.148 [0.098; 0.198]	0.357
IAA free	0.038 [0.026; 0.051]	0.075 [0.026; 0.124]	0.054 [0.025; 0.082]	0.377
HA total	2.029 [1.393; 2.666]	2.814 [1.665; 3.964]	2.730 [1.589; 3.871]	0.843
HA free	1.036 [0.681; 1.392]	1.692 [0.839; 2.544]	1.552 [0.891; 2.213]	0.710
CMPF	0.458 [0.307; 0.611]	0.775 [0.490; 1.060]	0.700 [0.355; 1.046]	0.160
UA	6.162 [5.591; 6.733]	6.565 [5.977; 7.152]	6.120 [5.613; 6.627]	0.545

Data are presented as median and confidence interval [CI]; patients were allocated to a high, moderate or low handgrip strength group based on the tertiles of the predicted handgrip strength, >103%, from 83 to 103%, and <83% respectively.

Table S4. Lower limb muscle strength and uremic toxins.

**Uremic Toxin Concentrations (mg/dL)**	**Quadriceps Muscle Strength**			***p*-Value**
	**High (>64%)**	**Moderate (46–64%)**	**Low (<46%)**	
IS total	1.235 [0.844; 1.625]	2.002 [1.552; 2.452]	1.970 [1.384; 2.555]	0.013
IS free	0.075 [0.046; 0.105]	0.146 [0.083; 0.209]	0.176 [0.085; 0.267]	0.026
pCS total	2.922 [2.307; 3.536]	3.899 [3.091; 4.707]	3.373 [2.658; 4.088]	0.218
pCS free	0.196 [0.144; 0.247]	0.312 [0.214; 0.409]	0.303 [0.172; 0.433]	0.193

pCG total	0.217 [0.117; 0.317]	0.335 [0.195; 0.474]	0.371 [0.205; 0.537]	0.301
pCG free	0.201 [0.105; 0.296]	0.312 [0.181; 0.443]	0.334 [0.182; 0.487]	0.279
IAA total	0.126 [0.092; 0.160]	0.179 [0.130; 0.229]	0.185 [0.101; 0.269]	0.096
IAA free	0.035 [0.023; 0.047]	0.054 [0.036; 0.072]	0.078 [0.023; 0.134]	0.076
HA total	2.081 [1.259; 2.903]	2.413 [1.659; 3.155]	3.078 [1.471; 3.166]	0.556
HA free	1.086 [0.616; 1.557]	1.332 [0.869; 1.795]	1.862 [0.928; 2.796]	0.397
CMPF	0.547 [0.264; 0.830]	0.631 [0.434; 0.421]	0.752 [0.422; 1.082]	0.181
UA	5.960 [5.474; 6.447]	6.559 [5.986; 7.133]	6.319 [5.720; 6.919]	0.240

Data are presented as median and confidence interval [CI]; patients were allocated to a high, moderate or low quadriceps muscle strength group based on the tertiles of the predicted quadriceps muscle strength, >64%, between 46 and 64%, and <46% respectively.

Table S5. Between-groups of functional prognosis analysis.

**Variable**	**Good Functional Prognosis (*n* = 32)**	**Poor Functional Prognosis (*n* = 43)**	***p* Value**
IS total	1.891 [1.469; 2.312]	1.621 [1.246; 1.996]	0.148
IS free	0.130 [0.082; 0.178]	0.134 [0.078; 0.190]	0.453
pCS total	3.463 [2.859; 4.066]	3.362 [2.792; 3.933]	0.855
pCS free	0.252 [0.183; 0.321]	2.834 [0.200; 0.367]	0.740
pCG total	0.247 [0.167; 0.327]	0.352 [0.230; 0.473]	0.793
pCG free	0.230 [0.154; 0.307]	0.321 [0.208; 0.434]	0.910
IAA total	0.180 [0.128; 0.232]	0.151 [0.108; 0.194]	0.265
IAA free	0.059 [0.034; 0.084]	0.053 [0.025; 0.081]	0.688
HA total	2.320 [1.642; 2.998]	2.660 [1.819; 3.502]	0.805
HA free	1.273 [0.851; 1.694]	1.529 [0.956; 2.092]	0.839
CMPF	0.651 [0.418; 0.884]	0.634 [0.428; 0.840]	0.915
UA	6.449 [5.941; 6.957]	6.159 [5.757; 6.561]	0.520

Data are presented as mean and confidence interval [CI]; patients were allocated to a good and poor function prognosis group based on the 300 m cut-off point on the 6MWT.
